# Supplementary material for: Epidemiological characteristics of imported acute infectious diseases in Guangzhou, China, 2005–2019
Source: PLoS Negl Trop Dis. 2022 Dec 6;16(12):e0010940. doi: 10.1371/journal.pntd.0010940 (PMC9725138; doi:10.1371/journal.pntd.0010940)
Supplement: Attachment — Submitted filename: point-to-point response to reviewers comments.docx [file pntd.0010940.s003.docx]

Dear Dr. Mallhi and Dr. Al-Salem,

Thank you for providing us with the opportunity to revise our manuscript entitled “**Epidemiological characteristics of imported acute infectious diseases in Guangzhou, China, 2005-2019**” (Submission ID: PNTD-D-22-00528). The reviewers’ comments were valuable and helpful in improving our paper. Accordingly, we have carefully revised the manuscript. We have uploaded two versions of the revised manuscript: one with tracked changes and the other a clean version.

We have addressed all concerns and comments raised by the reviewers as detailed in the response-to-reviewers letter. The revision mainly includes the following points:

1. We provided the definition and the calculation method of "Seasonal Index" in the section of method.
2. We added S2 Table to summarize the distribution of source (origin) for each imported infectious disease reported in Guangzhou.
3. We have given more discussion about how to strength the detection procedure for imported cases and why there was an exceptionally high peak of reported dengue cases in 2014.
4. We have worked on the text to improve clarity and acknowledge the study limitations highlighted by Reviewers

We would be very grateful if you could consider this paper for publication in *PLOS Neglected Tropical Diseases*.

Yours sincerely,

Chun-Quan Ou

School of Public Health, Southern Medical University

Guangzhou, China

Lei Luo

Guangzhou Center for Disease Control and Prevention,

Guangzhou, China

**Methods**
-Are the objectives of the study clearly articulated with a clear testable hypothesis stated?
-Is the study design appropriate to address the stated objectives?
-Is the population clearly described and appropriate for the hypothesis being tested?
-Is the sample size sufficient to ensure adequate power to address the hypothesis being tested?
-Were correct statistical analysis used to support conclusions?
-Are there concerns about ethical or regulatory requirements being met?

Reviewer #1: The importance and objectives of the study were nicely illustrated in the background section. As this is a retrospective study I think the study design was appropriate as expected in this type of epidemiological study. Sample size is alright considering the case definition and to draw the conclusions they did.

**[Response]:** We thank the reviewer for taking the time to evaluate our manuscript and for the positive assessment and valuable comments.

The statistical tests that has been done looks ok. Still, I think you should have mentioned the definition of "Seasonal Index" and how you calculated this in the method section.

**[Response]:** As suggested, we have described the definition in the revised manuscript as following: “Meanwhile, seasonal index was used to describe the seasonal fluctuation in the number of imported cases. The index for a given month was calculated by the average case number of that month divided by the average monthly cases during the 15 years (2005–2019).” (Page 7, Line 108-111)

Reviewer #2: The methodology in this study is not very clear and requires major revisions, which are suggested below.

**[Response]:** Thank you for these valuable suggestions and comments. The relevant section has been revised as the following point-to-point response.

Reviewer #3: The objectives are clear and appropriately addressed. Minor comments:
1. Please add 'Imported cases' as key words. And remove the word imported from 'imported acute infectious diseases'

**[Response]:** Thank you for this valuable comment and we fully agree with the reviewer about the adjustment of the key words. We have thus revised the key words.

2. Add the definition of imported case in the case definition section (with reference). Add the references for 'Acute infectious diseases'

**[Response]:** An **imported case** was defined as a patient who had visited or lived in an endemic or epidemic area outside mainland China within the longest incubation period of the acute infectious disease before the onset of symptoms [1]. **Acute infectious diseases** are characterized by symptoms of acute onset with a clear epidemiological exposure history [2]. We have added two references below in Line 95 and Line 99 in the revised manuscript.

References

[1] Wang Y, Wang X, Liu X, Ren R, Zhou L, Li C, et al. Epidemiology of Imported Infectious Diseases, China, 2005–2016. Emerg Infect Dis. 2018;25: 33–41. doi:10.3201/eid2501.180178

[2] Jiang L, Huang T. Comparison of the epidemiological aspects of acute infectious diseases between foreign and native imported cases in the border counties of Southwest China, 2008-2017. Epidemiol Infect. 2019;147: e230. doi:10.1017/S095026881900119

**Results**
-Does the analysis presented match the analysis plan?
-Are the results clearly and completely presented?
-Are the figures (Tables, Images) of sufficient quality for clarity?

Reviewer #1: The analysis presented looks good according to the analysis plan and are clearly stated in the result section.
I have few concerns for this section:
1) The figures provided are blur and not clear. Please provide a better version of each plot.

**[Response]:** To improve the quality of graphs, we have provided vector graphs instead of the original bitmaps. The compiled PDF file of submission may only provide the figures with low-resolution images shown after the reference list. A high-resolution version can be obtained by clicking the link at the top of each preview page.

2) Another analysis would be wonderful to include, which is a correlation analysis between major diseases and source (origin) of the disease. This analysis would be helpful for the government during management of situation or control the spread of the disease.

**[Response]:** Thank you for this nice suggestion. We have summarized the distribution of original countries of each imported infectious disease reported in Guangzhou (S2 Table).

3) In the supporting information file you provided a table containing "The number of reported cases of dengue in Guangzhou, 2005-2019". According to that, there was an exceptionally high peak of reported dengue cases in 2014 (i.e. 37359). But you did not explain whether this issue inside the paper. You should explain if this was caused by imported cases and how. And if this is completely domestic epidemic your statement in the discussion section (line 180-181) is not correct. Please explain.

**[Response]:** Sorry for the confusion caused. The statement in line 180-181 was cited from previous studies, which draw the conclusion through phylogenetic and phylogeographic analysis or epidemiological surveys [3,4]. The epidemic in 2014 was originated from imported cases followed by domestic extensive epidemic. We added S1 Table to present the number of total cases and the number of imported cases of dengue each year in Guangzhou. Although only 36 out of the total 37359 cases in 2014 were imported cases from outside China, many cases were domestically transmitted from the adjacent cities like Foshan, Zhongshan where the first dengue case in 2014 in these adjacent cities was also imported by oversea travel and then spread to nearby city such as Guangzhou [5]. Besides, extraordinary high precipitation in May and August, 2014 increased vector abundance, which means the environment can support more mosquitoes that transmit viruses [6]. After the unprecedented outbreak in 2014, the government paid more attention to early detection of imported cases, early mosquito control and the quarantine of every suspicious case, therefore, the outbreak size after 2014 was relatively small even the imported cases increased [6]. We have added the following sentence to explain these important points: “There was a high peak of reported dengue cases in 2014 in Guangzhou. Although only 36 out of the total 37359 cases in 2014 were imported cases from outside China (Table S1), many cases were domestically transmitted from the adjacent cities like Foshan, Zhongshan where the first dengue case in 2014 was also imported by oversea travel. Besides, extraordinary high precipitation in May and August, 2014 increased vector abundance, which means the environment can support more mosquitoes that transmit viruses. The government paid more attention to early detection of imported cases, early mosquito control and the quarantine of suspicious cases after the unprecedented outbreak in 2014. Therefore, the outbreak size after 2014 became relatively small even though the imported cases increased” (Page 11 Line 193-201)

References

[3] Sang S, Chen B, Wu H, Yang Z, Di B, Wang L, et al. Dengue is still an imported disease in China: A case study in Guangzhou. Infect Genet Evol. 2015;32. doi:10.1016/j.meegid.2015.03.005

[4] Jing Q-L, Yang Z-C, Luo L, Xiao X-C, Di B, He P, et al. Emergence of dengue virus 4 genotype II in Guangzhou, China, 2010: survey and molecular epidemiology of one community outbreak. BMC Infect Dis. 2012;12: 87. doi:10.1186/1471-2334-12-87

[5] Zhang Z, Jing Q, Chen Z, Li T, Jiang L, Li Y, et al. The increasing menace of dengue in Guangzhou, 2001–2016: the most important epicenter in mainland China. BMC Infect Dis. 2019;19: 1002. doi:10.1186/s12879-019-4504-3

[6] Cheng Q, Jing Q, Spear RC, Marshall JM, Yang Z, Gong P. Climate and the Timing of Imported Cases as Determinants of the Dengue Outbreak in Guangzhou, 2014: Evidence from a Mathematical Model. PLoS Negl Trop Dis. 2016;10: e0004417. doi:10.1371/journal.pntd.0004417

4) In the line 187 of the discussion section you mentioned "study years (0~17)". What does it mean. If this is a typo, please correct it.

**[Response]:** Sorry for this unclear description. “0~17” means the range of annual imported cases of malaria during the study period. We have re-written this sentence as following: “The number of imported malaria cases remained at a relatively low level in Guangzhou with the number of annual imported cases ranging from 0 to 17 during the study years.” (Page 11, Line 202-203)

Reviewer #2: Major revisions are required when it comes to the results section of the paper. The figures do not have any legends associated with them. The legends are mentioned in the results section instead.
**[Response]:** As suggested, we have added legends for the figures.

Reviewer #3: Good analysis. minor comment.
In table 2, add an additional column for other case.

**[Response]:** As suggested, we have added a column for other cases in **table 2**.

**Conclusions**
-Are the conclusions supported by the data presented?
-Are the limitations of analysis clearly described?
-Do the authors discuss how these data can be helpful to advance our understanding of the topic under study?
-Is public health relevance addressed?

Reviewer #1: Conclusions are mostly supported by the data presented. Authors also mentioned the major limitations of the study. They also discussed the public health importance and relevance.

**[Response]:** We thank the reviewer for the favorable comment.

Reviewer #2: The conclusion section does not have references and re-writing of the section is highly suggested.

**[Response]:** We only summarized the primary findings of this study in the conclusion section and therefore did not cite any references. We have re-written this section to give a clearer presentation.

Reviewer #3: limitations of analysis are clearly described.
Please add some points regarding strengthening of the detection procedure for imported cases.

**[Response]:** As suggested, we have discussed the importance and some recommended measures for the detection of imported cases. The revision is as following: **“**The screening and detection of imported infectious diseases should be strengthened and improved. Specifically, at first, it is essential to strengthen training of medical staff to improve the awareness and ability to identify and diagnose imported cases. Second, the customs authority should enhance the health monitoring and screening of people entering from endemic countries. If they have fever and other relevant symptoms, in addition to tests for SARS-CoV-2 infection, it is recommended to screen arthropod-borne infectious diseases such as dengue fever. Third, during the epidemic season of dengue fever in Guangzhou, rapid antigen testing can also be considered on inbound persons with a history of residence in the areas with a high prevalence of dengue fever, and the information should be promptly updated to the community for strengthening surveillance in their districts. Forth, it is also important to highlight the publicity and health education on common imported infectious diseases among entry-exit persons.” (Page 13, Line 234-245)

**Editorial and Data Presentation Modifications?**
Use this section for editorial suggestions as well as relatively minor modifications of existing data that would enhance clarity. If the only modifications needed are minor and/or editorial, you may wish to recommend “Minor Revision” or “Accept”.

Reviewer #1: Minor revision

Reviewer #2: Line 20 :- "...Southeast Asian..." should be re-written as "...Southeast Asia..."
Line 47 :- "Infectious diseases, which are known to have no boundaries..."
Line 49 :- Please replace "globally" with "worldwide".
Line 49 :- Please remove the word " worldwide".
Line 50 :- What do you mean by Chinese entry and exit? Please rewrite that.
Line 50-51 :- Please remove " mass population".
Line 54 :- "Increasing rapid and mass population movement"--very repetitive. Please rewrite that.
Line 55 :- Please re-write the entire sentence.
Line 55 :- What is the "Belt and Road" initiative?
Line 56 :- Please remove the word "importation".
Line 57 :- Can you elaborate with numbers, that the number of cases went up from 2005-2016?
Line 58-59 :- "The assessment.... measures"--please remove the line.
Line 59-60 :- Please explain the epidemiological characteristics of imported infectious diseases in China, in detail.
Line 62-64 :- Please elaborate.
Line 66 :- Please add a reference.
Line 68 :- Please replace "prone-prone"
Line 69 :- Please rewrite "...diseases in Guangzhou has not been investigated..."
Line 71 :- Why were these specific years 2005-2019 chosen for the study?
Line 72 :- The words "acute imported infectious diseases" ---very repetitive.
Line 86-87 :- Very repetitive. Please re-write.
Line 87 :- What is "unified national diagnostic criteria"?
Line 92 :- The words "acute imported infectious diseases" ---very repetitive.
Line 101-102 :- Please rewrite.
Line 156-157 :- The words "acute imported infectious diseases" ---very repetitive.
Line 157 :- "...Guangzhou were mainly seen in males, aged 20-49 years..."
Line 160-161 :- Please elaborate this sentence.
Line 163 :- Please add a reference.
Line 198 :- Please add a reference.

**[Response]:** As suggested, the minor revisions have been made in the revised manuscript.

Reviewer #3: (No Response)

**Summary and General Comments**
Use this section to provide overall comments, discuss strengths/weaknesses of the study, novelty, significance, general execution and scholarship. You may also include additional comments for the author, including concerns about dual publication, research ethics, or publication ethics. If requesting major revision, please articulate the new experiments that are needed.

Reviewer #1: I think the major weakness of the study is that it is based on the symptomatic cases only. However, it was a well-organized manuscript with clear objectives, and I think, the authors have are mostly successful to achieve their goal.

**[Response]:** It is indeed the limitation of this study. The cases reported in the China Information System for Disease Control and Prevention, including laboratory-confirmed cases and clinically diagnosed cases, were diagnosed by clinicians according to the diagnostic criteria enacted by the Ministry of Health of the People’s Republic of China. The asymptomatic cases cannot be detected or reported, leading to underestimation of imported cases. This highlights the importance of developing and implementing relevant procedures and measures for rapid and early detection of imported cases including asymptomatic cases to control the transmission of imported diseases. We have discussed this issue in the revised manuscript. (Page 13, Line 240-244)

Reviewer #2: (No Response)

Reviewer #3: The manuscript is nicely presented. Figures are appropriate.

PLOS authors have the option to publish the peer review history of their article ([what does this mean?](https://journals.plos.org/plosntds/s/editorial-and-peer-review-process#loc-peer-review-history)). If published, this will include your full peer review and any attached files.
If you choose “no”, your identity will remain anonymous but your review may still be made public.
**Do you want your identity to be public for this peer review?** For information about this choice, including consent withdrawal, please see our [Privacy Policy](https://www.plos.org/privacy-policy).

Reviewer #1: No

Reviewer #2: No

Reviewer #3: No
